# A Case of Adult-Onset Periodic Fever, Aphthous Stomatitis, Pharyngitis, and Cervical Adenitis (PFAPA) Syndrome Responsive to Tonsillectomy in Japan

**DOI:** 10.1155/2019/1746180

**Published:** 2019-09-16

**Authors:** Kohei Yamahara, Yuki Egawa, Kana Lee, Noriyuki Nakashima, Satoshi Ikegami

**Affiliations:** ^1^Department of Otolaryngology, Head and Neck Surgery, Shizuoka City Shizuoka Hospital, Shizuoka, Shizuoka 420-8630, Japan; ^2^Department of Pathology, Shizuoka City Shizuoka Hospital, Shizuoka, Shizuoka 420-8630, Japan; ^3^Department of Otolaryngology, Shin-Suma General Hospital, Kobe, Hyogo 654-0048, Japan; ^4^Department of Physiology, Kurume University School of Medicine, 67 Asahi-Machi, Kurume 830-0011, Japan

## Abstract

Adult-onset periodic fever, aphthous stomatitis, pharyngitis, and cervical adenitis (PFAPA) syndrome is a rare condition, having been reported in only three patients in Japan till date. While almost all pediatric PFAPA patients respond well to tonsillectomy, some European studies have reported that tonsillectomy may be ineffective for adult-onset PFAPA. All the Japanese patients with adult-onset PFAPA had been treated orally so far (cimetidine with or without prednisone), instead of tonsillectomy. We reported a case involving a 37-year-old Japanese man with PFAPA syndrome who presented with a history of febrile episodes associated with pharyngitis, cervical adenitis, and aphthous stomatitis for one year. The patient had been undergoing oral medication therapy without any significant improvement. Tonsillectomy was performed for the patient, and complete resolution of PFAPA was achieved. Our experience suggests that a tonsillectomy is a viable option for the treatment of adult-onset PFAPA.

## 1. Introduction

Periodic fever, aphthous stomatitis, pharyngitis, and cervical adenitis (PFAPA) syndrome is the most common autoimmune inflammatory fever disorder of childhood worldwide [1]. It is characterized by high periodic febrile episodes that last for approximately four days and are associated with pharyngitis, cervical adenitis, and aphthous stomatitis [2]. The underlying etiology of the disease is still unknown, and the diagnosis is made using the clinical criteria [2, 3]. PFAPA is generally considered to be a condition specific to the pediatric population; however, some European studies have reported adult-onset PFAPA cases ever since the first adult case of this disease was observed in 2008 [3]. Adult-onset PFAPA is still a rare entity in Japan, and only three Japanese patients with adult-onset PFAPA have been reported so far [4–6]. All these three patients were being treated with oral medication (cimetidine with or without prednisone), instead of undergoing tonsillectomy. While almost all pediatric PFAPA patients respond well to tonsillectomy, the effect of this procedure in adult-onset PFAPA has not been determined as yet. Here, we report a case of a Japanese patient suffering from adult-onset PFAPA syndrome, which was refractory to oral medication therapy but showed a favorable response to tonsillectomy.

## 2. Case Presentation

A 37-year-old man was referred to the Otolaryngology, Head and Neck department in Shizuoka City Shizuoka Hospital with a history of episodic recurrent febrile illness. He led an active life till the age of 36 years, when he first developed a high fever (39°C) associated with a sore throat, aphthous stomatitis, and enlargement of the bilateral cervical lymph nodes with associated tenderness. These episodes always resolved spontaneously after about five days, regardless of therapy with nonsteroidal anti-inflammatory drugs or antibiotics. Thereafter, the patient experienced similar recurrent febrile episodes every 4^th^ week. Initially, he was seen by his physician and was thought to have a viral infection and tonsillitis, which was being managed with antibiotics. However, the patient continued to experience similar attacks at regular intervals, and they were so regular that he could foresee the exact day when the next episode would occur.

On examination, the patient was not in any apparent distress and looked well-built. Of the laboratory tests performed, only a slightly elevated level of C-reactive protein was observed. The immunoglobulin and serum complement levels were within normal limits, and the patient tested negative for immune-phenotypic markers of lymphocytes, HIV, CMV, EBV, and antinuclear antibodies. Cyclic neutropenia was excluded by serial neutrophil counts. Bacterial cultures from a throat swab and blood sample showed no growth. Computed tomography (CT) revealed enlargement of the bilateral submandibular lymph nodes with no evidence of other abnormal findings. Transthoracic echocardiogram revealed no bacterial vegetations. His family history was unremarkable, and no mutations were detected in the genes responsible for the most commonly found hereditary periodic fevers (HPF), familial Mediterranean fever (FMF), and TNF receptor-associated periodic syndrome (TRAPS) on genetic testing.

PFAPA syndrome was eventually diagnosed according to Padeh's criteria (Table 1), after a single dose of 60 mg oral prednisone resulted in abrogation of the episodes, leading to a marked improvement in the patient's general well-being and return of the body temperature to normal. To prevent future febrile episodes, we prescribed oral medication, cimetidine 800 mg daily and oral prednisone 60 mg at the time of the febrile attack. We continued the oral medication for 6 months; however, the febrile episodes kept recurring, and rather, the attacks became more frequent and occurred every 2^nd^ week even after starting oral treatments.

Since oral therapy was ineffective, we performed a tonsillectomy for the patient. On the next day after the procedure, the patient experienced high fever (39°C) that continued for two days, after which the body temperature spontaneously returned to normal. The postoperative course was uneventful, and no recurrences of febrile episodes were observed during the follow-up of two years postoperatively although the patient was not taking any oral medication. The patient provided written informed consent for the publication of this case report.

## 3. Discussion

We report a case of adult-onset PFAPA syndrome that was refractory to oral medications but responded to tonsillectomy completely. To the best of our knowledge, this is the first reported case of the PFAPA syndrome in an adult Japanese patient, who was successfully treated with tonsillectomy.

Although the pathogenesis of PFAPA has not been well elucidated to date, it is considered an autoinflammatory disease [7, 8]. An increasing number of patients, including Japanese individuals, are being diagnosed as adult-onset PFAPA [3–6, 9], and the clinical characteristics of adult-onset PFAPA seem to be similar to those of pediatric-onset PFAPA. To date, the treatment options for both pediatric and adult-onset PFAPA include oral steroids, cimetidine therapy, or tonsillectomy [9]. Corticosteroids have been successfully used during febrile episodes and can dramatically reduce fever attacks in a few hours. Shortening of the symptom-free interval has been previously reported with the use of steroids, which was similar to what we observed in the present study [10]. However, steroid administration does not prevent further attacks [11, 12].

Cimetidine is the drug of choice for preventing recurrent febrile attacks, but low remission rates have been reported (27%) in pediatric patients following its use [13]. There are only a few reports on the efficacy of cimetidine therapy for adult-onset PFAPA. In the previous cases of Japanese adult-onset PFAPA, including the present case, cimetidine was administered in 4 patients, with a complete response seen in 2 patients and ineffectiveness of the drug observed in 2 patients.

Although tonsillectomy is not always helpful, complete resolution of pediatric-onset PFAPA after tonsillectomy has been reported in several case series and in two randomized trials, suggesting that the tonsils are the primary site of immune dysregulation in PFAPA [14–16]. On the other hand, some European studies reported that tonsillectomy was not effective for adult-onset PFAPA patients [9, 17]. Cantarini et al. reported that only 2 out of 9 patients who underwent tonsillectomy showed a clinical response [9]. Alam and Hammoudeh reported a case of adult-onset PFAPA where the patient was treated with tonsillectomy that led to a decrease in severity and frequency of episodes, which lasted only for 6 months [17]. Thereafter, this patient again experienced regular febrile episodes in the form of fever, aphthous stomatitis, and pharyngitis [17]. In our report, tonsillectomy completely resolved the symptoms in an adult-onset patient (no symptoms were observed at a follow-up at two years postoperatively), just as in pediatric-onset PFAPA.

Although careful observation is needed, our case demonstrated that tonsillectomy could be an effective option for the treatment of adult-onset PFAPA. There is no convincing evidence to explain why tonsillectomy is effective for some patients and not for other patients. Histological analysis of the tonsils from adult PFAPA patients might provide answers to this question because some previous studies have revealed unique histological features of the tonsils from children with PFAPA [18, 19]. If some histological differences are detected between the tonsils of patients in whom tonsillectomy was effective and those in whom tonsillectomy was ineffective, it might help clinicians understand the prognosis of future patients of adult-onset PFAPA who might require a tonsillectomy.

The three previously reported Japanese patients with adult-onset PFAPA were treated by rheumatologists, not otolaryngologists. However, PFAPA patients are likely to consult otolaryngologists first because their symptoms mainly manifest in the ears, nose, and throat. Adult-onset PFAPA is still a rare entity in Japan, but increased awareness of PFAPA will result in an increased number of adult-onset PFAPA patients. Although cooperation between otolaryngologists and physicians or rheumatologists is warranted, it is more essential for all otolaryngologists to familiarize themselves with the clinical symptoms of adult-onset PFAPA and take the lead in the treatment of this disease.

## 4. Conclusion

Tonsillectomy is a viable and effective option for the treatment of adult-onset PFAPA although further studies should be conducted to investigate the outcome and therapeutic options for this disease in finer detail. Increased awareness of clinical PFAPA will result in an increased number of adult-onset PFAPA patients. Considering that PFAPA patients are likely to consult specialists first, this study implies that otolaryngologists should take the lead in the treatment of PFAPA in cooperation with physicians and rheumatologists.

## Figures and Tables

**Table 1:** Diagnostic criteria of PFAPA.

Monthly fevers—cyclic fever at any age group
Exudative tonsillitis with negative throat culture
Cervical lymphadenitis
Possibly aphthous stomatitis
Completely asymptomatic interval between episodes
Rapid response to a single dose of corticosteroids
